# Modeling microbiota-associated human diseases: from minimal models to complex systems

**DOI:** 10.20517/mrr.2022.01

**Published:** 2022-05-13

**Authors:** Doriane Aguanno, Amira Metwaly, Olivia I. Coleman, Dirk Haller

**Affiliations:** ^1^Chair of Nutrition and Immunology, Technical University of Munich, Freising 85354, Germany.; ^2^ZIEL Institute for Food & Health, Technical University of Munich, Freising 85354, Germany.

**Keywords:** Human microbiota-associated mouse models, fecal microbiota transplantation, inflammatory bowel diseases, obesity, metabolic diseases, colorectal cancer, host-microbiota interactions

## Abstract

Alterations in the intestinal microbiota are associated with various human diseases of the digestive system, including obesity and its associated metabolic diseases, inflammatory bowel diseases (IBD), and colorectal cancer (CRC). All three diseases are characterized by modifications of the richness, composition, and metabolic functions of the human intestinal microbiota. Despite being multi-factorial diseases, studies in germ-free animal models have unarguably identified the intestinal microbiota as a causal driver of disease pathogenesis. However, for an increased mechanistic understanding of microbial signatures in human diseases, models require detailed refinement to closely mimic the human microbiota and reflect the complexity and range of dysbiosis observed in patients. The transplantation of human fecal microbiota into animal models represents a powerful tool for studying the causal and functional role of the dysbiotic human microbiome in a pathological context. While human microbiota-associated models were initially employed to study obesity, an increasing number of studies have applied this approach in the context of IBD and CRC over the past decade. In this review, we discuss different approaches that allow the functional validation of the bacterial contribution to human diseases, with emphasis on obesity and its associated metabolic diseases, IBD, and CRC. We discuss the utility of simple models, such as *in vitro* fermentation systems of the human microbiota and *ex vivo* intestinal organoids, as well as more complex whole organism models. Our focus here lies on human microbiota-associated mouse models in the context of all three diseases, as well as highlighting the advantages and limitations of this approach.

## INTRODUCTION

The human body harbors several hundred different microbial species, which collectively encode about 150-fold more genes than those in the human genome^[1-3]^. The microbiota encompasses bacteria, viruses, fungi, and archaea that inhabit different niches in the human body and have coevolved with humans over the past six million years to establish a tightly regulated symbiotic relationship. The intestinal microbiota contributes to the regulation of epithelial cell homeostasis and barrier integrity^[4]^, the maturation and differentiation of the mucosal immune system^[5]^, and the coordination of systemic metabolic and endocrine functions^[6]^. The disruption of mutualistic microbiome-host interactions in the intestine drives tissue and organ aberrations, which may lead to the initiation or progression of diseases. The change in intestinal microbiota composition (dysbiosis) has been implicated in a wide range of chronic diseases, including metabolic disorders (e.g., obesity and obesity-associated metabolic diseases including type 2 diabetes mellitus (T2DM) and non-alcoholic fatty liver disease (NAFLD)^[7-11]^, immune-mediated diseases [such as inflammatory bowel diseases (IBD)]^[12-17]^, and colorectal cancer (CRC)^[18-20]^.

Obesity and its associated-metabolic diseases, IBD, and CRC are recognized as multi-factorial diseases with a globally rising disease incidence^[21-23]^. Their etiology involves a complex interaction of genetic, environmental, and immune-mediated factors^[24-29]^. The three disease entities share a common basis of chronic inflammation associated with dysbiotic intestinal bacterial communities, characterized by reduced richness, in addition to a reduction of beneficial microbes and an expansion of putative pathobionts^[30]^. Alterations in intestinal bacteria composition have been described in obesity and T2DM. Previous reports showed an increased abundance of *Escherichia coli*,* Veillonella*,* Blautia*,* Anaerostipes*,* Lactobacillus*, *Faecalibacterium*, and Clostridiales in T2DM. On the contrary, a reduced abundance of *Bacteroides*,* Bifidobacterium*,* Parabacteroides*, *Oscillospira *and the mucin-degrading *Akkermansia muciniphila* was shown to be associated with improved metabolic health^[31-33]^. Similarly, in IBD, inflammatory responses in humans as well as experimental mouse models are linked to the over-representation of certain pathobionts such as *Clostridium*, *Fusobacterium*, Segmented Filamentous Bacteria (SFB), adhering invasive *Escherichia coli *(AIEC), and *Enterococcus faecalis*^[12,15,34]^ and the reduction in beneficial butyrate-producing bacteria, such as *Ruminococcacea*e and *Lachnospiraceae*^[12,15,35-37]^. Additionally, the gut microbiota in patients with CRC shows an imbalanced bacterial community composition, characterized by a significant increase in *Bacteroides fragilis*, *Fusobacterium nucleatum*, *Campylobacter*, *Enterococcus faecalis*, and *E. coli* and a decrease in butyrate-producing *Faecalibacterium*, *Blautia*, *Clostridium*, and *Roseburia*^[38,39]^. Notably, patients with early-stage colorectal tumors (advanced adenomas) were shown to have a different gut microbiota composition compared to those with late-stage tumors (CRC)^[40]^, suggesting that a dysbiotic gut microbiota plays a role in tumor progression.

**Figure 1:**
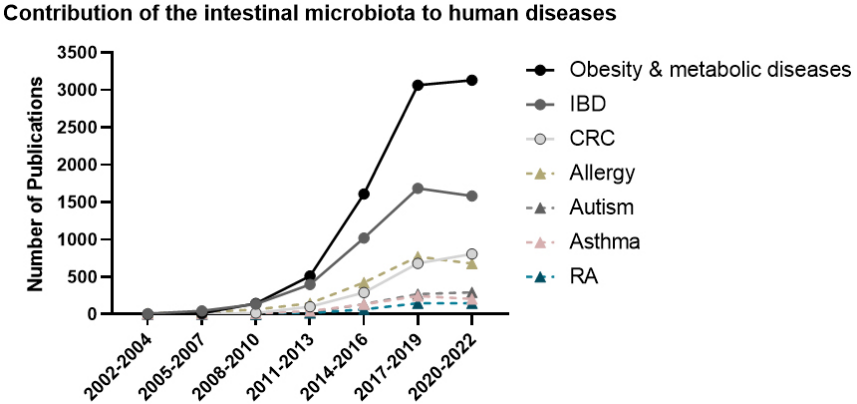
The number of publications related to the contribution of the intestinal microbiota to human diseases in the last two decades, grouped per year. Data were obtained by searching PubMed (https://pubmed.ncbi.nlm.nih.gov/) with the term “gut microbiota”, and each human pathology indicated (retrieved 21 December 2021): Obesity, type 2 diabetes (T2D), inflammatory bowel disease (IBD), colorectal cancer (CRC), asthma, allergy, rheumatoid arthritis (RA), and autism.

Multiple lines of evidence unarguably identify the intestinal microbiota as one of the non-genetic central factors causally driving pathogenesis of these three diseases, as illustrated by the number of publications during the last decade [Figure 1]. In this regard, both host-microbe and microbe-microbe interactions form critical components of disease progression (pathobionts) as well as disease prevention (protective bacteria), depending on the specific microbe and disease. In metabolic diseases, germ-free (GF) mice are leaner than conventional mice and resistant to weight gain on a high-fat diet^[41,42]^. However, this protection against weight gain has been shown to be diet-dependent and not only rely on the microbiota presence^[43,44]^. Similarly, intestinal inflammation only develops in the presence of bacteria in most experimental models of IBD, whereas animals housed under GF conditions remain disease-free^[45-50]^. Equally, the observed absence of or reduction in tumor formation in CRC mouse models housed under GF conditions or subjected to antibiotic treatment clearly identifies the intestinal microbiota as a key driver in CRC initiation, progression, and metastasis^[51-53]^. Dissecting the underlying mechanisms of host-microbiota interactions in disease onset and progression is indispensable and requires representative models that mimic the complexity of the human gastrointestinal tract, including the intestinal epithelium, the gut microbial communities, and the immune milieu. At present, experimental human microbiota-associated mouse models are of great relevance in dissecting the complex interplay between microbes and the genetically susceptible host. Human microbiota-associated animal models using a human-derived microbiota of different complexities (complex, minimal consortia, or a single strain) allow us to uncover mechanisms of pathogenesis, as well as microbe-host interactions in numerous human pathologies, including obesity and its associated-metabolic diseases, IBD, and CRC^[10,18,54-72]^. Despite the known limitations of human microbiota transfer into GF mice^[73]^, gnotobiotic models enable the study of perturbations in the gut microbiota in a controlled experimental setup, allowing the assessment of causality of the complex host-microbiota interactions. Next to *in vivo *animal models, *in vitro* fermentation systems have proved to be particularly useful in simulating human gut physiology through the rigorous control of experimental conditions, such as bacterial community density, luminal redox and pH, and gut transit time, which is not feasible using *in vivo *models*. *In addition, advancements in organoid microfluidics technology and three-dimensional *ex vivo* models of the human intestinal epithelium facilitate the study of the complex interactions between the microbiota and the intestinal epithelium and allow the discovery of novel metabolites as microbial targeting therapies. 

In this review, we critically discuss the utility of different *in vitro, ex vivo,* and *in vivo *model systems in regard to the study of host-microbe interactions, focusing on the microbiota. We discuss the advantages and disadvantages of these models, with a particular focus on human microbiota-associated (HMA) mouse models, using obesity and its associated-metabolic diseases, IBD, and CRC as examples of three widely studied disease entities.

## THE SIMPLER MODELS

### *In vitro* fermentation

Using mouse models in gut microbiome research profoundly enhanced our understanding of complex microbe-host interactions in the pathogenesis of IBD, obesity and its associated metabolic diseases, and CRC^[74-76]^. However, translating the results from mouse models to humans remains challenging due to intrinsic anatomical, physiological, and ecological differences between the two systems that need to be considered^[77,78]^. To bridge this gap, continuous *in vitro* fermentation of complex gut microbial communities has been successfully developed to investigate microbe-microbe interactions while reducing animal testing and circumventing host confounding factors^[79-84]^. These models enable the cultivation of human-derived fecal samples under simulated physiological conditions (e.g., retention time, temperature, pH, and redox potential) that simulate the spatial, environmental, and temporal features of specific ecosystems, enabling translational mechanistic studies^[85]^. The complexity of *in vitro* fermentation systems ranges from simple batch culture systems to continuous *in vitro* fermentation systems (single-stage, multistage, or artificial fermentation)^[86-89]^. Each of these models has advantages and disadvantages, and the selection of the appropriate model depends largely on the study objectives.

The simplest model is batch fermentation, in which a pure or mixed bacterial community is grown in a selected medium inside sealed reactors or bottles under anaerobic conditions. This model is suitable for short-term experiments due to the rapid substrate depletion and reduction of pH, which can prevent further microbial activity. Simple batch fermentation is particularly useful for dietary compound fermentation studies^[90-92]^. For instance, simple *in vitro* batch fermentation was used to evaluate the prebiotic effect of new dietary components through testing the effect of the prebiotic on ammonia concentration, pH, and short chain fatty acid (SCFA) production in fecal cultures^[90]^. Furthermore, an *in vitro* batch fermentation model of the human colon can be used to replicate the microbial metabolic pathways in humans and thus stand as a suitable model for studying bacterial metabolism and for screening potential therapeutic targets^[93]^. Complex questions of bacterial metabolic modulation require continuous fermentation models where substrate replenishment and toxic product elimination are ensured under the tight control of growth conditions. Single-stage models are often used to mimic the proximal colon conditions. Conversely, multistage continuous fermentation allowed a more precise simulation of human colonic function that differs along the colonic regions (in bacterial composition and their metabolic activity), by combining three chemostats connected in series, replicating the proximal, transverse, and distal colon regions^[94,95]^. Artificial digestive systems have been developed to simulate the human gastrointestinal tract as well as its digestive functions. For instance, SHIME (simulator of the human intestinal microbial ecosystem) combines a series of five fermentation vessels that are operated in sequential batch mode, with the first two reactors simulating the digestive processes in the duodenum/jejunum and ileum, which are connected to the three-stage large intestinal model^[96]^. A major limitation of *in vitro* fermentation models is the inadequacy of simulating the host functionality (e.g., lack of immune milieu). To overcome this limitation, fermentation models incorporating intestinal cell cultures (e.g., colon epithelial cell cultures and/or immune cells) are implemented to reproduce the host responses *in vitro*^[97]^.

Considering the pros and cons of each approach, complementing animal models and human studies with *in vitro* fermentation studies would broaden our insights into the complex relationship among the gut microbiota, diet, and host^[83]^.

### *Ex vivo* intestinal organoids

Three-dimensional cellular models better mimic intestinal architecture and physiology and have overtaken cell lines, which are usually derived from cancerous cells and display modified characteristics in molecular pathways. The group of Hans Clever published two pioneer studies showing the formation and growth of small intestinal^[98]^ and colonic^[99]^ organoids from intestinal epithelial stem cells, characterized by their 3D structure organized in proliferative crypt-like compartments and differentiated intestinal epithelial cell (IEC) types forming a monolayer. Additionally, organoids can be generated from induced pluripotent stem (iPS) cells and minced tissue^[100]^.

The capacity of organoids to self-organize in compartments mimicking the intestinal epithelial structure (proliferative crypts and villi containing differentiated cell types), the presence of nearly all cell types forming the intestinal epithelium, and the possibility to grow them from various mice genotypes or human donors make them powerful tools to study intestinal homeostasis and model diseases. Monitoring the growth and differentiation of organoids in real time provides insight into cell death/proliferation rates in a dynamic way, which would not be possible *in vivo*. For example, Nagpal *et al.* showed that the abundance of Firmicutes and Verrucomicrobia in Leptin-deficient mice positively correlated with an abnormal cellular turnover, while Bacteroides species abundance negatively correlated with these markers of epithelial homeostasis in organoids derived from Lep^ob/ob^^[101]^.

As an intermediate model between cell lines and mouse models, organoids allow the modeling of intestinal diseases with genetic modifications, notably in the study of genes of which the knockout causes embryonic lethality, while also reducing the use of animals. The ability to culture human organoids derived from donors has enabled us to better characterize the epithelial contributions to intestinal diseases. IBD-related changes in DNA methylation identified in IEC were reproduced and maintained in pediatric IBD donor-derived organoids^[102]^, as well as patient-specific abnormal epithelium polarity, proliferation properties, and inflammation levels^[103]^. Reproducing genetic mutations *ex vivo* also allowed for deciphering the consequences of somatic mutations found in IBD^[104]^. Intestinal organoids have been particularly useful to recapitulate phenotypes of CRC patient tumors. Noteworthy, colorectal organoid “living” libraries, including rare clinical subtypes, have been established and showed the importance of niche factors^[105]^, as well as the possibility for applying high-throughput drug screening^[106,107]^, thus emphasizing organoids as a tool allowing personalized medicines. Interestingly, very few studies investigate the epithelial contribution to obesity and metabolic diseases employing intestinal organoids. Nonetheless, Hasan *et al.* demonstrated that human intestinal organoids retained the glucose absorption characteristics of obese donors^[108]^. In addition, a study showed a proof of principle that human intestinal organoids (derived from iPS) can be modified and used as surrogate glucose-responsive and insulin-producing cells, which survived *in vivo *in mice. Altogether, these works highlight the potential of organoids in understanding human diseases and complementary therapeutic approaches.

Despite their above-mentioned advantages, organoid applications in the field of intestinal diseases display critical limitations. The lifespan of organoids in culture-in the range of weeks-restricts studies to short-term effects and constrains experimental setups to passaging to prevent the overgrowth of organoids, which would eventually lead to cell death. In addition, heterogeneity in size, shape, and differentiation of organoids generates variability within and between experimental conditions and can hinder possible readouts, such as growth and budding measurements. Conversely, this diversity and plasticity may highlight important morphological and functional differences, notably in human organoids. Exemplary, IBD patient-derived organoids were shown to exhibit various structural epithelial phenotypes based on the donor and the level of inflammation^[103]^. Donor-to-donor heterogeneity and variability can be assessed and characterized to produce donor-specific data profiles^[109]^. Studying cellular mechanisms is possible by adjusting the growth factors in the media to modify the composition in stem cells *versus *differentiated cell lineages in organoids^[110]^ and even further to favor specific rare epithelial cell types^[111]^. However, organoids do not recapitulate the level of complexity of the intestinal environment, which results from intricate interactions among the epithelial cells, immune cells, nervous system, and, importantly, commensal microorganisms.

New techniques to “engineer” organoids, such as air-liquid interface and “organ-on-a-chip”^[112]^, provide tools to circumvent the absence of a mesenchymal compartment, vascularization, and the intestinal microbiome, through co-cultures with other cell types, notably immune cells, and have been reviewed by others^[113,114]^.

When it comes to studying direct host-microbiota interactions, the co-culture of intestinal organoids with bacteria faces the technical challenge of bacteria accessing the apical epithelium to mimic the physiological polarized interaction, while the basolateral side is easily reachable for the addition of, e.g., cytokines. Such technical pitfalls, outlined in more detail below, might be one of the major limitations of applying *ex vivo* organoids in the microbiome field to study microbiota-associated human diseases. Besides fragmentation or direct addition of bacteria or their products to the media, microinjection of bacteria into the lumen was the first method of accessing the apical side of the organoids^[115]^. The technical difficulties of this method (low number of replicates, clogging of the micropipette, and contamination due to organoid breaching) have led to the development of alternative approaches, such as the seeding of organoids as a 2D monolayer on semi-permeable filters (with distinct and easily accessible apical and basolateral compartments)^[114]^ or the novel method of forcing apical-out polarity^[116]^. While the first approach allows the maintenance of organoids in culture over a longer period of time, it lacks the 3D architecture. The second technique is physiological but does not fully recapitulate *in vivo* tissue structure (as apical-out organoids are mostly cystic) and has the disadvantage of a very short lifespan in culture (up to five days). Finally, and more importantly, aerobic growth conditions suited for organoids challenge the survival of bacteria, notably commensal intestinal anaerobic bacteria. This methodological aspect is particularly critical in the field of host-microbiota interactions. Nonetheless, recent progress has been made to overcome this problem. A recent study showed an engineered physiodynamic system with an anoxic-oxic interface that allows the co-culture of the human microbiome (shown to form microcolonies) with organoids derived from patients with Crohn’s disease, ulcerative colitis, or CRC and exhibiting disease-specific differentiations^[117]^. Another recent study also used a microfluidic platform to co-culture human donor-derived colonic organoids in monolayers with the super oxygen sensitive bacterium *Faecalibacterium prausnitizii*^[118]^. This system, anoxic on the apical side and oxic in the basolateral compartment, allowed for cultivating both bacteria and human cells for four days. These engineered platforms open perspectives to extend organoid methods to microbiota-host interactions in a physiological way.

Taken together, *ex vivo* organoids constitute a useful model to decipher molecular and cellular mechanisms in complement to more complex setups such as whole organisms. This relatively recent approach will continue to evolve technically, bringing complexity and refining the possibilities to answer questions related to host-microbiota interactions.

## COMPLEX *IN VIVO* MOUSE MODELS

Experimental mouse models are valuable tools to study the functional impact of the gut microbiota on host health, thus helping to study basic immunological and microbe-host mechanisms of multiple human diseases^[119-121]^. Key advantages of using mouse models in studying human disease include low cost, availability of a wide range of inbred strains, and ease of genetic manipulation to represent certain aspects of the clinical phenotype or underlying mechanism of the human disease. At present, well-controlled animal facilities possess specific pathogen-free (SPF) mouse husbandry, where the mice are free of known pathogens but present with an indigenous microbiota of undefined composition. The exclusion of pathogens ensures increased uniformity and reproducibility of research results^[122]^. Although animal models can be informative, they fail to mimic the human gut microbiome and thus have limited translational potential for human microbiota-associated diseases. Therefore, disease-relevant HMA mouse models have been successfully established through the transfer of human microbiota into GF mice by fecal microbiota transplantation (FMT). A few limitations have been reported for HMA, including the incomplete maturation of the host immune system due to the absence of the microbiota during early life, leading to an impaired sensitivity of the immune system to inflammation^[123,124]^. Alternatively, microbiota depletion following antibiotic treatment has been used by some researchers to overcome these limitations^[125-127]^. Unlike GF animals, the antibiotic treatment allows the study of the role of gut microbiota in adult mice while maintaining cell functionality and immune system development^[128-130]^. In one study, the pre-treatment of mice with a four-day course of ciprofloxacin followed by daily inoculation of human donor microbiota through oral gavage was successful in establishing only a fraction of the complex bacterial community. Interestingly, a more extensive regimen of five different antibiotics, namely amphotericin-B, vancomycin, neomycin, metronidazole, and ampicillin, improved the engraftment efficiency of the transplanted human microbiota. However, this protocol required more extensive exposure to human microbiota through weekly gavage for 12 consecutive weeks^[127]^. As such, antibiotic treatment offers an inexpensive and less demanding alternative to GF mice; however, they still have the limitation of incomplete depletion of microbes and potential off-target effects, which might impact mitochondrial ribosomes and protein synthesis processes^[131,132]^ and hence impact the findings of these experiments. For example, bactericidal antibiotics have been shown to disrupt the mitochondrial electron transport chain, leading to the buildup of reactive oxygen species in mice treated with clinically relevant doses of bacteriostatic antibiotics^[133]^. To improve the translational modeling of disease, one group established a new mouse model that acquired the microbes and pathogens of wild mice while maintaining the genetic background of the laboratory mice. This approach is known as “wildling”. Notably, in two pre-clinical studies, the wild gut microbiota promoted host fitness and improved resistance to influenza A virus pulmonary infection^[134,135]^ and inflammation-induced CRC^[136]^.

### Human microbiota-associated mouse models

**Figure 2:**
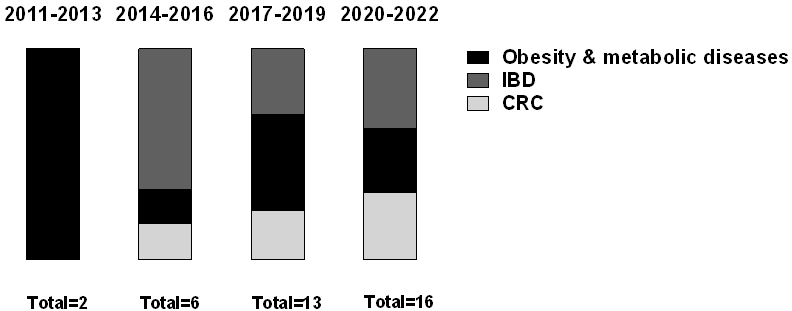
The number of publications using human microbiota-associated mouse models to study the contribution of the intestinal microbiota to IBD, obesity and its associated metabolic diseases, and CRC in the last decade grouped per year. Data were obtained by searching PubMed (https://pubmed.ncbi.nlm.nih.gov/) with the term “human fecal microbiota transplantation” or “colonized germ-free” or “Human microbiota-associated mice” and each human pathology indicated (retrieved 21 December 2021): obesity, inflammatory bowel disease (IBD), and colorectal cancer (CRC).

To characterize the extent to which dysbiotic bacterial communities have a functional impact on disease pathogenesis, multiple studies utilized HMA mouse models, where GF or antibiotic-treated mice are colonized with single, simplified, or complex human-derived bacterial populations. The HMA mouse model proved to be extremely useful in testing the functional impact of colonization with putative pathobionts on the host disease phenotype and immune response. In the quest to understand the role of dysbiosis in human diseases, studies using HMA mouse models to recapitulate obesity have been pioneer works^[63,137]^. Further studies followed to extend this model to IBD and CRC [Figure 2]. Although HMA mice are widely used to address the role of the gut microbiome in disease causality, these models have a number of evolutionary, ecological, and methodological limitations that can impact the interpretation of the data, as reviewed previously^[73,138]^. In the following sections, we review and critically discuss HMA mouse model studies that highlight the role of the human microbiota in obesity and its related metabolic diseases, IBD, and CRC (for a summary, see Table 1).

**Table 1:** Use of human microbiota-associated mouse models in modeling microbiota-linked human diseases

**Disease**	**Recipient**	**Colonization/Experiment**	**Human disease recapitulation & outcome**	**Reference**
Obesity (2009)	GF C57BL/6J male mice	Donor: human fecal sample from a single healthy donor Methodology: single oral gavage of either fresh or frozen sample diluted in PBS. Diet switch from a low-fat, plant polysaccharide-rich diet to a high-fat, high-sugar diet	Humanized mice fed the Western diet have increased adiposity Transmissibility of phenotype by microbiota transplantation	[137]
Obesity (2013)	GF C57BL/6J male mice	Donor: human microbiota from four twin pairs discordant for obesity, or with culture collections from an obese (ob) or lean (Ln) co-twin Methodology: single oral gavage. Mice were fed a chow diet or one of two diets (high or low in saturated fats)	Obesity and metabolic phenotype were transmissible to humanized mice Co-housing of (ob) and (Ln) rescued mice from the phenotype	[63]
Obesity (2014)	GF Swiss Webster mice	Donor: human fecal microbiota from twin pairs with detectable methanogens (L+), lean without methanogens (L-), obese with methanogens (O+), or obese without methanogens (O-) Methodology: single oral gavage with fecal samples: Donor stool Donor stool amended with a heat-killed *C. minuta* Donor stool amended with live *C. minuta*	*Christensenella abundances* were higher in the L+ group and lower in the O- group, mirroring the family *Christensenellaceae* enrichment in lean compared to obese human individuals When donor stool lacking detectable* Christensenella *was amended with* C. minuta*, recipient mice had lower weights and reduced adiposity compared to unamended stool-transferred animals	[56]
Obesity and metabolic phenotype (2017)	GF Swiss Webster mice	Donor: fecal microbiotas from 16 obese children/adolescents and 16 matched controls Methodology: single oral gavage (2 animals/donor sample)	Weight gain in mice colonized with microbiotas from obese donors (after 7 days and up to 52 days post-inoculation). Positive correlation between weight gain in recipients and fat percentages of the human donors. The mice microbiotas gradually became more similar to the original human inoculum over time Microbiotas of the recipient mice differed among the groups, although the fecal microbiotas of human donors were not different. Spread of microbial species between cages within isolators	[67]
Obesity and NAFLD (2018)	C57BL/6J male mice	Donor: stool samples from 2-week-old infants born to normal weight (Inf-NWMB) or obese mothers (Inf-ObMB), based on pre-pregnancy BMI Methodology: a single oral gavage (2 animals/donor samples) with pooled stool samples from 2-3 infants from each group	Increase in subcutaneous white adipose tissue (but not in total body weight), disrupted intestinal barrier function and pro-inflammatory state of the liver in Inf-ObMB-colonized mice compared to Inf-NWMB mice. The Inf-ObMB phenotype was exacerbated by a 6-week western-style diet The differences between groups observed in the infant cohort from which the samples were taken were recapitulated in animals after transfer. After 6 weeks of western-style diet feeding, the differences in composition and metabolism of the gut microbiota were abolished	[64]
Obesity (2019)	GF C57BL/6N male mice	Donor: 2 obese individuals with high relative levels of *Clostridium ramosum* Methodology: oral inoculation with human fecal mixture and 4-week colonization phase. Immunization with microbial antigens, CpG oligodeoxynucleotides, and/or curdlan (3 groups: naïve, CpG + curdlan, and CpG + curdlan + antigen). After 2 weeks, switch to a high-fat diet (HFD)	Induction of *Clostridium ramosum*-specific IgG and IgA. Lower *C. ramosum* levels in the colonic mucosa of vaccinated mice compared to those of naïve mice and mice immunized without antigen Mice vaccinated with antigen showed significantly lower weight gain, lower epididymal and mesenteric white adipose tissue, decreased Slc2a2 levels in the ileal epithelium, and blood glucose levels than control animals, despite similar food intake, suggesting a preventive effect of vaccination on *C. ramnosum*-mediated obesity	[70]
Obesity (2019)	GF C57BL/6J male mice	Donor: adult female dizygotic twins discordant for obesity (1 lean and 1 obese individual in total) Methodology: oral gavage with a consortium of bacterial strains cultured from a fecal microbiota sample	Animals colonized with lean twin-microbiota had significantly higher VO_2_ per lean body mass (LBM), which is a major contributor to energy expenditure, compared to obese co-twin microbiota-associated animals. Fat mass was reduced in mice inoculated with lean *vs. *obese microbiota, despite no differences in body weight and food intake	[71]
Obesity (2020)	C57BL/6J male mice	Donor: obese and non-obese donors divided into 2 groups based on cognitive scores Methodology: oral gavage of fecal samples after 14 days of antibiotic cocktails. Booster inoculations twice per week throughout the study	Memory scores from human donors matched with respective recipient animals. Bacterial species from the donor’s microbiota, such as *Akkermansia sp*., *Subdoligranulum sp.*, *Clostridium*,* Ruminococcus*, and *Roseburia sp*., were associated with increased memory scores of recipient mice, while several *Bacteroides sp*. were negatively associated with this score. Mice inoculated with microbiota from non-obese donors showed increased memory scores compared with obese donors’ microbiota-colonized animals	[69]
Obesity and insulin resistance (2021)	SPF- or conventional- housed C57BL/6J mice	Donor: three lean (Ln) and three obese (Ob) donors Methodology: engraftment of human fecal microbiota from Ln or Ob human donors in mice housed under SPF or conventional conditions through a single oral gavage after 7-day antibiotic conditioning. Study limited by a small number of animals	The human obese phenotype was transmitted to conventional mice, but not SPF animals, when the engraftment was successful and with a donor-specific phenotypic response. The efficiency of the engraftment was donor-dependent, irrespective of housing and sex. Relative abundances of *Bacteroidia *and* Gammaproteobacteria* classes in the donor sample correlated with 1-week engraftment efficiency, while *Clostridia *negatively correlated with 1-week engraftment	[57]
Obesity-mediated vascular dysfunction and glucose intolerance (2021)	GF C57BL/6J mice	Donor: lean or obese donors, selected on their endothelial dysfunction (obese) Methodology: a single oral gavage of fecal inoculate	Induction of vascular dysfunction and glucose intolerance in GF mice colonized with obese donors’ microbiota compared to lean, despite no differences in body and tissue weight between animal receivers of lean *vs.* obese microbiota	[72]
NAFLD (2021)	GF or SPF male C57BL/6N mice	Donor: individuals with high brown adipose tissue BAT (*n* = 3) or low BAT (*n* = 4) Methodology: 8-week colonization period after a single gavage with fecal material	No differences in results between GF- or SPF-colonized mice. The transmission of the human microbiota did not lead to alterations in body mass, fat mass, or BAT activity in the recipient mice	[68]
Inflammatory Bowel Diseases (2016)	GF C57BL/6 and *Il10^-/-^* mice	Donor: human microbiota from healthy donors and CD or UC patients Methodology: FMT via oral gavage into 8-16-week-old GF C57BL/6 mice for 2 weeks (HM repository mice). Fecal samples isolated from HM repository mice were inoculated into GF* l10^-/-^* mice for 3 weeks	CD microbiota induced more severe colitis than healthy control microbiota IBD-associated microbiota induced pro-inflammatory gene expression profile that resembles signatures found in CD patients	[61]
Inflammatory Bowel Diseases (2019)	GF C57BL/6J and C57BL/6J Rag1-deficient (B6.129S7-Rag1tm1Mom/J) mice	Donor: stool samples from healthy or IBD donors Methodology: FMT via oral gavage at 4-6 weeks of age for 4 weeks	IBD microbiotas increased numbers of intestinal Th17 cells and Th2 cells and decreased numbers of RORγt+ Treg cells in comparison to healthy donors in colonized mice The proportions of induced Th17 and RORγt+ Treg cells were predictive of human disease status in the Rag1*^-/- ^*colitis model	[55]
Inflammatory Bowel Diseases (Crohn’s disease) (2019)	GF ATG16L1 T300A knock-in mice (4-weeks association)	Donor: patient stool from Crohn’s disease patients with a genotype of WT or T300A and with active or inactive disease status Methodology: FMT via oral gavage (25 mg = 100 µL) followed by 12.5 mg (50 µL) placed on the anus and 12.5 mg (50 µL) placed on the back fur of each mouse	Mutant showed an increased abundance of *Bacteroides* and elevated intestinal Th17 cells than the WT mice None of the mice developed intestinal inflammation, suggesting that changes to gut bacteria and immune response may precede disease incidence	[58]
Inflammatory Bowel Diseases (2020)	GF C57/Bl6	Donor: stool samples from CD mother-baby pairs and from control mother-baby pairs Methodology: FMT via oral gavage at 6-8 weeks of age for 5 weeks	GF mice inoculated with the stool of mothers with CD and their 3-month-old babies have significant abnormalities in the adaptive immune cells compared with mice inoculated with stool from non-CD controls	[65]
Inflammatory Bowel Diseases (2020)	GF WT 129Sv/Ev and *Il-10^-/-^* mice	Donor: patient stool from Crohn’s disease patients at baseline and during remission or relapse following hematopoietic stem cell transplantation (HSCT) Methodology: FMT via oral gavage from 8 to 12 weeks of age	CD fecal transplant transfers disease states in *Il-10^-/-^* mice, while WT mice remained disease-free Sulfur metabolism links disease activity to human microbiome in humanized mice	[60]
Colorectal Cancer (2014)	GF + AOM/DSS C57/BL6	Donor: fecal samples from 3 healthy controls and 3 CRC patients Methodology: FMT followed by AOM i.p after 3 weeks and 3 cycles of DSS for 5 days	Non-invasive adenomas with dysplastic changes. Variation of tumor number linked to initial inoculum differences, not CRC status Potential tumorigenic role of Bacteroidales and protective role of certain members of Crostidiales. Tumor incidence linked to butyrate production and host glycan degradation	[18]
Colorectal Cancer (2017)	SPF + Abx + AOM C57/BL6 GF C57/BL6	Donor: pooled stool samples from 5 healthy controls or 5 CRC patients Methodology: SPF, 2 weeks of Abx *ad libitum*, followed by AOM i.p. and twice-weekly FMT for 5 weeks; GF, one-time FMT at 8 weeks	SPF: CRC FMT induced colonic polyps with high-grade dysplasia and histological inflammation, an increase in tumorigenesis-associated genes, and significantly lowered bacterial richness CRC FMT increased proliferation and showed an increase in inflammation-associated genes and immune cell infiltration in GF recipient mice	[66]
Colorectal Cancer (2019)	SPF + Abx Apc^min/+^ SPF + Abx C57/BL6	Donor: pooled fecal samples from 10 healthy controls or 10 CRC patients Methodology: 3 days of Abx *ad libitum*, followed by twice-weekly FMT for 8 weeks	Enhanced progression of intestinal adenomas in Apc^min/+^ mice following CRC patient FMT CRC stool induced chronic low-grade inflammation and intestinal mucosal barrier damage	[59]
Colorectal Cancer (2021)	GF C57/BL6	Donor: patient stool from healthy or CRC who were high or low in rice bran consumption Methodology: FMT at 8-10 weeks, followed by AOM i.p. and three cycles of 2% DSS for 5 days	Neoplastic lesions in the intestine Rice bran-modified microbiota causes fewer neoplastic lesions in the colon	[62]

GF: Germ-free; NAFLD: non-alcoholic fatty liver disease; BMI: body mass index; SPF: specific pathogen-free; BAT: brown adipose tissue; CD: Crohn’s disease; UC: ulcerative colitis; FMT: fecal microbiota transplantation; IBD: inflammatory bowel diseases; WT: wild type; AOM: azoxymethane; DSS: dextran sodium sulfate; CRC: colorectal cancer.

### HMA mice in obesity and obesity-associated metabolic diseases

In the last 50 years, obesity has become a major public health concern worldwide, with nearly 40% of the world’s adult population estimated to be overweight in 2016 [body mass index (BMI) of ≥ 25 kg/m^2^], of whom over 10% were affected by obesity (BMI ≥ 30 kg/m^2^), representing a prevalence rate three times higher than in 1975^[139]^. The role of the intestinal microbiota in metabolism was assessed for the first time using GF rodent models in a historical 1983 study^[140]^. A couple of decades later, the pioneer group of Jeffrey Gordon at Washington University showed more detailed mechanisms of the contribution of the presence of intestinal microbiota to fat storage and obesity onset in GF mice^[41,42]^. These studies initially focused the scientific interest regarding the contribution of the microbiota to diseases on obesity and thus led to more rapid progress in this field compared to IBD and CRC, as illustrated in Figure 2. Further studies followed to assess specifically the role of the human microbiota in these metabolic diseases, which are discussed below.

In 2009, Turnbaugh *et al*. investigated, in one of the earliest HMA studies, the effect of a high-fat/high-sugar Western diet on human microbiota transferred into GF recipient mice^[137]^. The switch from a low-fat, plant polysaccharide-rich (LF/PP) diet to a Western diet rapidly induced changes in the microbiota composition within a single day with an increased abundance of bacteria belonging to the *Erysipelotrichi *class (Firmicutes phylum) and in particular in organisms related to *Clostridium innocuum*,* Eubacterium dolichum*, and *Catenibacterium mitsuokai*. HMA mice fed a Western diet had increased adiposity, and this feature could be transmitted to new recipients transplanted with cecal samples from the HMA mice. Interestingly, while the human microbiota could be further transferred to the second generation of animals, the authors showed that a switch in the diet of recipient animals quickly abolished the legacy effects participating in shaping the initial bacterial community (depending on the mouse donor). Of note, the engraftment of the human microbiota was successful and stable even after four weeks. While this study showed interesting results regarding methodology when comparing fresh or frozen samples and transmission of the microbiota to second-generation donors, it was only performed using a single human donor and the human donor effect could not be considered.

To decipher the microbiota’s contribution to the complex and multi-factorial etiology of obesity, independently of genetic factors, several groups compared HMA effects from lean or obese co-twins. A 2013 study showed the effect of the human microbiota from obese or lean twins on mouse body phenotype, as obese HMA animals had higher adiposity than lean HMA animals^[63]^. Furthermore, the microbiota in mice displayed functional differences between obese and lean donors, being more prone to polysaccharide breakdown and fermentation. Interestingly, the authors observed the same metabolic consequences when the human microbiota was cultured *in vitro* prior to inoculation. The increase in adiposity could be rescued when co-housing animals with lean HMA mice, and the most prominent invading species were *Bacteroides cellulosilyticus*,* Bacteroides vulgatus*,* Bacteroides thetaiotaomicron*,* Bacteroides caccae*,* Bacteroides uniformis*,* Alistipes putredinis*, and *Parabacteroides merdae*. The presence of the last three Bacteroidetes species positively correlated with cecal acetate, propionate, and butyrate levels^[63]^. In line with this, a method study aiming to develop a calorimetry approach in a GF isolator setting showed a higher energy expenditure and reduced fat mass in animals colonized with the microbiota from a lean co-twin compared to the obese sibling^[71]^. Conversely, another group used over 100 fecal samples from UK twin pairs to investigate the role of host genetics on the gut microbiota^[56]^. The authors showed that monozygotic twins had a more highly correlated microbiota composition compared to dizygotic twin pairs and identified a co-occurring network formed by the family *Christensenellaceae *with associated methanogenic Archaea as a heritable taxon, which were enriched in lean individuals. When transplanting the human microbiota from lean donors in the presence or absence of methanogens (respectively, L+ or L-) or from obese donors (O+ or O-), the abundance of *Christensenellaceae *correlated with a lower body weight gain. The addition of *Christensenella minuta* to the stool reduced the weight gain in recipient mice^[56]^.

Two studies investigated the role of the microbiota from younger subjects on obesity, but with different outcomes. A 2017 study showed that the obese phenotype from children or adolescents could be recapitulated in GF animals, which exhibited a more similar microbiota composition to the donor with time^[67]^. However, the microbiota profiles were different between mouse groups, unlike human samples, and microbial species spread between cages^[67]^, showing the complexity of experimentally mimicking the human microbiome. Soderborg and colleagues highlighted for the first time the role of the mother’s microbiota early colonization of the intestine in newborn infants^[64]^. GF mice associated with stool samples from two-week-old infants born to obese mothers (Inf-ObMB) displayed an increase in subcutaneous white adipose tissue, a disrupted intestinal barrier function, a pro-inflammatory state of the liver, and histological profiles typical of pediatric NAFLD, compared to mice colonized with stool samples from two-week-old infants born to normal-weight mothers (Inf-NWMB). This study emphasized the causal role of the microbiota in early events of metabolic diseases^[64]^. In contradiction to these findings, Ahmed and colleagues investigated the link between a lower brown adipose tissue (BAT) activity in adults with NAFLD and the gut microbiota, showing that BAT activity was not linked to the fecal microbiota and was not transmissible to mice colonized with the fecal microbiota from high or low BAT-activity donors^[68]^.

To expand these findings beyond obesity to other metabolic diseases, a recent study also investigated the contribution of an obesity-associated microbiota to the development of metabolic disorders such as vascular dysfunction and glucose intolerance^[72]^. Interestingly, both the vascular dysfunction and glucose intolerance could be transmitted to obese-donor HMA GF mice but not to lean-donor HMA mice, despite similarities in body and tissue weights between recipient groups.

As mentioned above, studies using HMA models in the scope of obesity are multiple and comprise various contexts, as illustrated hereafter. Fujimoto and colleagues investigated the implication of the obesity-associated bacterium *Clostridum ramnosum* on the onset of the disease and showed that vaccination with *C. ramnosum* antigen prevented the development of the obese phenotype^[70]^. In a different context, Arnoriaga-Rodríguez *et al*. demonstrated that obesity was associated with impaired memory, and, interestingly, transferring the microbiota of obese donors resulted in lower memory scores in HMA mice^[69]^.

The choice of model and experimental conditions can affect the results and outcomes of HMA mouse studies, as presented below. A recent study compared the effect of housing conditions on the transmission of the disease phenotype and showed that the obese phenotype could only be transmitted to animals raised under conventional housing and not SPF, both pre-treated with antibiotics^[57]^. While this last study only included a small number of animals, it raises the question of experimental procedures and housing conditions when investigating the recapitulation of multi-factorial diseases by HMA models. Supporting this idea, a recent study showed that mice hosting a wild microbiota (“wildlings”) were protected from weight gain when fed a high-fat diet (HFD), unlike SPF mice^[141]^. As this resistant phenotype could only be observed in animals exposed to the wild microbiota in their early life and not during adulthood, this emphasizes the mature development of the intestinal tissue in other models such as SPF or GF.

More generally, the interpretation of changes in obese phenotype and microbiota profile must take into account experimental variables such as variations in mice strains, age and sex, and methodology in phenotype assessment (total body weight, dissection, and measurements of adipose tissue)^[142]^. Additionally, the nature of the high-fat diet has been shown to lead to diet-induced obesity independently of the microbiota^[43,44]^; the composition of control diet also impacts the outcomes of investigations, such as microbiota and metabolic changes^[143]^.

### HMA mice in IBD

Gnotobiotic mice of different bacterial complexity have been used to study the impact of IBD-associated dysbiosis on disease pathogenesis. For example, mono-colonization with *Bacteroides thetaiotaomicron* induced the expression of genes associated with host immune system maturation, including Treg activation^[144]^. Further, we previously showed through the mono-colonization of IL-10-deficient mice with a single strain of *E. faecalis *that the presence or absence of distinct virulence traits potentially modulates the colitogenic effect of this pathobiont in IBD^[49,145]^. The generation of disease-relevant minimal bacterial consortia enabled the study of more complex disease mechanisms. A simplified human microbiota consortium, which comprises seven bacterial strains isolated from patients with IBD, was shown to drive inflammation in the IL-10-deficient colitis mouse model through Th1 and Th17 cell responses and the successful colonization by the consortium members; AIEC and *Ruminococcus gnavus* were proven necessary for the induction of intestinal inflammation^[146]^. In a recent study, we characterized the colitogenic activity of *E. faecalis* as part of the SIHUMI consortium and demonstrated that complex microbe-microbe interactions can reprogram the colitogenic activity of *E. faecalis *toward a protective function, where the presence of *E. faecalis* was important for the upregulation of genes involved in growth and replication. In contrast, colonization with the SIHUMU consortium that lacks *E. faecalis* induced enhanced inflammation in mice^[50]^. The value of gnotobiotic mouse models has been further demonstrated through numerous FMT studies, in which fecal samples from IBD patients were transplanted into GF recipient mice. In one study, GF IL-10-deficient mice were colonized with IBD or healthy-associated microbiota. The HMA mice largely reflected the dysbiotic features (e.g., lower community richness and diversity) and the disease phenotype (inflamed or non-inflamed) observed in their respective human donors. Likewise, FMT of IBD-associated microbiota specifically induced pro-inflammatory immune responses that were lacking following the transplantation of a healthy microbiota. Notably, IBD or healthy HMA mice showed distinct luminal metabolic profiles, emphasizing the usefulness of HMA as a tool to study the functional impact of the microbiota on host immunity^[61]^. In a further study, Britton *et al*. examined the generalized immune response to FMT of human microbiota derived from healthy or IBD donors into ex-GF mice^[55]^. FMT of IBD microbiota into C57/BL6 GF mice induced greater induction of Th2 and RORγt^+^ (retinoid-related orphan receptor-γ short isoform) Th cells and reduced induction of RORγt^+^ T regulatory (Treg) cells, relative to FMT with the microbiota of healthy donors^[55]^. Notably, the proportions of microbiota-induced Th17 and RORγt^+ ^Treg were predictive of human donor disease status upon the transfer of IBD microbiota into *Rag1*-deficient mice. In addition to demonstrating the successful recapitulation of human disease in gnotobiotic mice, this study demonstrates that an IBD-associated microbiota is consistently more pro-inflammatory than that of healthy donors^[55]^. In contrast, Lavoie *et al.* exposed GF wild-type and ATG16L1T300A mutant mice (ATG16L1T300A, a gene that increases the risk of Crohn’s disease in humans) to human stools from patients with Crohn’s disease (CD)^[58]^. An increased level of *Bacteriodes *and Th17 cells was observed in mutant mice compared to WT mice. However, none of the mice developed intestinal inflammation^[58]^. A further study by Torres *et al.* investigated the effect of IBD-associated maternal and infant microbiota on the host immune system^[65]^. C57/BL6 GF mice were transplanted with stool from CD mothers or their respective three-month-old babies^[65]^. Additionally, three groups of mice were transplanted with stool from non-CD, control pregnant women or their three-month-old babies. GF recipients of IBD FMT showed reduced frequencies of homeostatic Treg and IgA^+^ B cell subsets compared to GF recipients of healthy control FMT. Collectively, this study demonstrated that maternal IBD-associated dysbiosis is transmissible to the offspring and leads to dysfunctional mucosal immunity lacking key homeostatic elements^[65]^. In addition to these reports, we recently used a toolbox of multi-omics analyses in an integrated framework, including 16S rRNA gene sequencing, shotgun metagenomics, and targeted and untargeted metabolomics together with HMA mice to characterize bacterial community structure, functional capability, and metabolic activity and to dissect microbe-host interactions of disease pathogenesis and the risk of relapse in a cohort of CD patients undergoing hematopoietic stem cell transplantation^[60]^. Shared fecal microbiome and metabolome signatures correlated with disease activity and recapitulated the disease state when transferred to GF mice. The experimental validation in HMA mice allowed the identification of sulfur metabolism as a key mechanism linked to disease activity in Crohn’s disease^[60]^.

### HMA mice in CRC

CRC is the second most common cause of cancer deaths worldwide, showing alarming progression in Western countries and increasing incidence in young adults^[147]^. To our knowledge, only four HMA mouse model studies of CRC have been conducted, three of which relied on the chemical induction of neoplastic lesions using azoxymethane (AOM)/dextran sodium sulfate (DSS). In the first of these three studies, Baxter *et al.* transplanted GF C57/BL6 mice with fecal microbiota from three separate CRC patients and three healthy controls, followed by AOM/DSS intervention after three weeks^[18]^. While this experimental setup successfully induced non-invasive adenomas with dysplastic changes, differences in phenotype severity were associated with baseline microbiome structures in recipient mice and not with the donor cancer status. Dirichlet multinomial mixture modeling of baseline communities identified three enterotypes associated with tumor susceptibility. Analysis of the community structure and inferred metagenome positively correlated Gram-negative *Bacteroides*, *Parabacteroides*, *Alistipes*, and *Akkermansia* and the potential for host mucin-glycan degradation with tumor burden, while Gram-positive *Clostridiales* and the capacity for SCFA butyrate production negatively correlated with tumor numbers. A second study investigated the effect of FMT of pooled CRC patient stool (*n* = 5) *versus* pooled healthy control stool (*n* = 5) into both GF and SPF-housed C57/BL6 mice^[66]^. GF mice received a one-time gavage at the age of eight weeks, while SPF mice were subjected to two weeks of antibiotic treatment, followed by a single dose of AOM and subsequent twice-weekly gavages of microbial communities for five weeks. GF recipients of CRC human microbiota showed an increased epithelial cell proliferation compared to GF recipients of healthy HMA. Furthermore, significantly higher proportions of SPF mice receiving CRC FMT presented with high-grade dysplasia and macroscopic polyps. Both GF and SPF recipients of CRC FMT showed decreased bacterial richness, an increase in tumorigenesis- and inflammation-associated genes, and immune cell infiltration compared to healthy HMA controls. One important aspect of the study design between these two described investigations is the FMT sample itself. While one study performed three separate CRC patient FMT associations, the other pooled CRC patient stool from five patients for a single application. In light of the large inter-individual differences observed in human microbial communities and the lack of a clearly defined CRC microbial signature, the selection of patient stool for HMA mouse models of CRC (or other microbiota-related diseases) is not trivial. In a third study, Parker *et al.* incorporated nutritional intervention into the investigation of microbiota-driven CRC and examined the neoplastic potential of human microbiota derived from CRC survivors, comparing those who consumed rice bran daily for 28 days [rice bran-modified microbiota (RMC)] and those who had no dietary intervention^[62]^. Colonization of selected microbiota inoculums into C57/BL6 GF mice subjected to AOM/DSS treatment revealed that RMC induced fewer neoplastic lesions in the colon. This protection was associated with enrichment in *Flavonifractor* and *Oscillibacter* and an increase in the anti-cancer metabolites myristoylcarnitine and palmitoylcarlnitine. In addition to presenting an HMA CRC model of successful transfer of disease phenotype, this study additionally highlights that nutritional intervention with rice bran can modify the intestinal microbiota to ameliorate CRC by improving metabolism.

It is important to consider that models of spontaneous CRC are favorable over chemically induced models, and that the AOM/DSS model, while closely mimicking the progression of tumors in CRC, creates a setting that recapitulates colitis-associated cancer rather than a classical CRC. The adenomatous polyposis coli (APC) gene knockout model (Apc^min/+^) model is a widely used murine model that spontaneously develops multiple intestinal adenomas with or without low-grade dysplasia^[148]^. Li *et al.* performed FMT of pooled fecal samples from CRC patients (*n* = 10) or healthy controls (*n* = 10) into SPF-housed Apc^min/+^ recipient mice or C57BL/6 controls following antibiotic intervention^[59]^. Recipient mice were inoculated with stool samples twice weekly for a period of eight weeks. While the disease was not recapitulated in C57BL/6J control mice gavaged with CRC patient stool, an enhanced progression of intestinal adenomas accompanied by chronic low-grade inflammation and mucosal barrier damage was observed in Apc^min/+^ mice^[59]^. In line with observations by Baxter *et al.*^[18]^, this study observed that CRC patient FMT increased the abundance of the mucin-degrading specialist *Akkermansia*, while reducing the abundance of SCFA-producing bacteria *Ruminococcus* and *Roseburia*^[59]^. The disadvantage of Apc mutation rodent models for CRC studies is the lack of tumor formation in the colon, which would warrant the use of spontaneous murine models with specific tumor formation in the colon, such as the transgenic nATF6^IEC^ murine model^[53]^. In these mice, chronic activation of the endoplasmic reticulum unfolded protein response (UPR^ER^) transcription factor activating transcription factor 6 (ATF6), specifically in intestinal epithelial cells (IECs), causes spontaneous colonic tumors in a microbiota-dependent and inflammation-independent manner^[53]^. For reliable identification of the causal role of a CRC-associated microbiota and functional validation of microbial signatures, extensive research using such models is warranted. 

### HMA models: limitations and methodological considerations

While studies on human cohorts provided great insights into the relevance of gut microbiome dysbiosis in multiple human diseases, the huge inter-individual variations among human subjects because of various genetic or environmental exposures (e.g., diet, medication, and geographical location) pose challenges in identifying specific disease-driving or -associated microbiome signatures^[149-151]^. Furthermore, most of the available patient cohorts are retrospective studies looking into the microbial shifts following rather than preceding disease onset, suggesting that these findings could be mere associations rather than causal changes linked to disease pathogenesis. HMA mouse models are considered an excellent means of addressing causality in disease pathogenesis and understanding complex host-microbe interactions. Moreover, HMA mice allow the application of integrated multi-omics analyses to identify functional causal links to disease development^[60]^. However, it is important to acknowledge these models have limitations considering the evolutionary, anatomical, and ecological differences between mice and humans^[3]^. One of the limitations lies in the bacterial transfer and engraftment efficiency from human to mouse. We previously showed that, while most of the top abundant taxa were transferred from IBD patient-derived microbiota to GF mice, their proportions changed substantially^[60]^. For example, the proportion of Bacteroidetes showed to increase in HMA mice compared to the original human donor^[127,152]^. In contrast, *Bifidobacterium spp*. and *Lactobacilli spp*. failed to be sufficiently engrafted in HMA mice, suggesting a selective pressure of the host habitat^[153,154]^. Besides, it is difficult to replicate the ecological factors associated with human disease development such as diet, geography, and lifestyle in HMA mice, making it difficult to translate the findings to the human scenario. Previous reports have additionally shown that HMA mice have a defective immune maturation and present clear differences in metabolic activity^[153,155]^.

Given these limitations of HMA mice, a few considerations have been suggested to increase the rigor of HMA experiments and thereby establish true causal links between the microbiome and disease^[73,78]^. The use of an appropriate number of human donors in HMA mice is extremely important to account for the huge variations seen in the human gut microbiome. As such, the number of human donors should account for the true biological replication rather than the number of recipient mice (to avoid pseudoreplication). The storage and cryopreservation of human fecal samples in glycerol for further transfer into GF mice is important to maintain the viability of bacteria and thus ensure successful bacterial engraftment. In-depth analysis of the successfully engrafted bacterial taxa and the functional impact on the host metabolism through integrated multi-omics analysis is important to identify microbiome signatures indicative of disease phenotypes. Finally, validation of the causal link between specific disease-associated bacterial taxa and disease development through mono-association studies is essential to establish causality between the microbiome and host phenotype.

## CONCLUDING REMARKS

**Figure 3:**
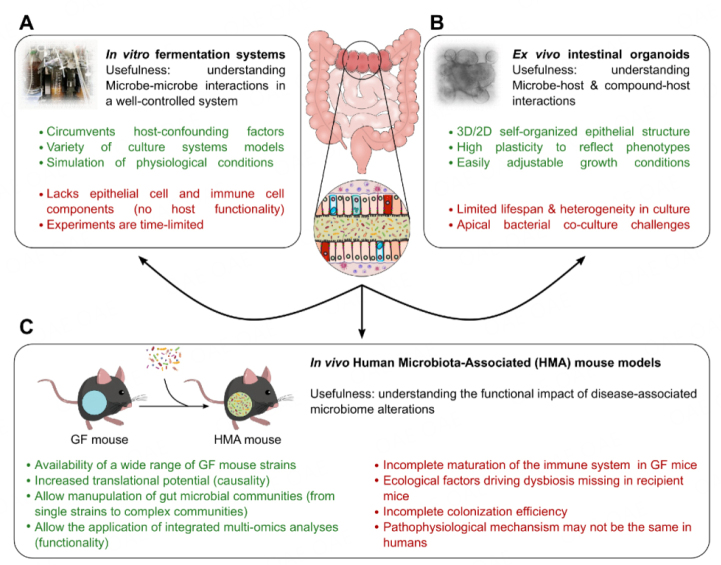
Preclinical models for the study of human microbiome-associated diseases. Overview schematic summarizing the different models of choice to study functional host-microbiota interactions, with their advantages and disadvantages, in human diseases of the digestive system, such as obesity and related metabolic disorders, inflammatory bowel diseases, and colorectal cancer. Depicted are: (A) *in vitro* fermentation systems; (B) *ex vivo* intestinal organoids; and (C) *in vivo* HMA mouse models. GF: Germ-free.

Over the last two decades, scientific interest in gut microbiota has increased significantly, and the link between the gut microbiome and numerous human diseases, including metabolic and inflammatory diseases and cancer, has been established. While most available microbiome studies reveal an association of dysbiotic gut bacterial communities with disease development, the underlying microbe-host interactions remain largely unclear. The wide array of available preclinical models allows tackling this aim through different approaches, addressing different aspects of the question. *In vitro* fermentation systems provide a tool to study microbe-microbe interactions in a host-independent manner;* ex vivo* organoid models allow us to decipher molecular mechanisms; and *in vivo* mouse models bridge all actors (the epithelium, immune system, and microbiota) of the disrupted host-microbiota interactions in pathological models. HMA mouse models have proved to be successful in recapitulating complex human diseases, such as obesity and its associated metabolic diseases, IBD, and CRC, and identifying potential mechanisms of microbe-host interactions. Considering the advantages and disadvantages of each modeling approach [Figure 3], complementing human studies with preclinical *in vitro* fermentation studies, *ex vivo* organoid systems, and *in vivo* HMA mouse models could help disentangle the complex interactions between the gut microbiota and the host.

## DECLARATIONS

### Copyright

© The Author(s) 2022.
